# The measles epidemic trend over the past 30 years in a central district in Shanghai, China

**DOI:** 10.1371/journal.pone.0179470

**Published:** 2017-06-22

**Authors:** Jie Gao, Bing Shen, JianJing Xiong, Yihan Lu, Qingwu Jiang

**Affiliations:** 1 The Key Laboratory of Public Health Safety of Minister of Education-School of Public Health, Fudan University, Shanghai,China; 2 Jing’an District Center for Disease Control and Prevention, Shanghai, China; Fudan University School of Public Health, CHINA

## Abstract

**Background:**

Measles vaccination over the past 50 years has greatly reduced the incidence of measles. However, measles among migrants and the resulting changes in epidemiological characteristics have brought new challenges to the elimination of measles. We aim to describe the measles epidemic trend over the past 30 years in a central district in Shanghai, China.

**Methods:**

The present study was conducted in the Jing’an District, which is located in the center of Shanghai. Based on historical surveillance data of measles, we calculated the incidence of measles among local residents and migrants separately. Next, we classified all of the cases of the measles among local residents between 1984 and 2015 into 8 age groups and 5 birth cohorts. Finally, we calculated the measles incidence in each time period by the different age groups and birth cohorts, to understand the measles epidemic trend over past 30 years in the Jing'an District.

**Results:**

A total of 103 cases of measles were reported from the Jing’an District, Shanghai, from 1984 to 2015. For infants less than 1 year of age and adults over 30 years of age, the incidence of measles continued to rise over the past 30 years. For a specific birth cohort, the incidence of measles after measles vaccination declined initially, and was then followed by a rebound.

**Conclusions:**

The incidence of measles in older adults and infants increased in some developed regions, which slows the process of measles elimination. This suggested that the population immunity against measles after measles vaccination would gradually reduce with time. We recommend supplemental immunization against measles in adults in order to reduce the immunity decline, especially for migrants.

## Introduction

**Fig 1:**
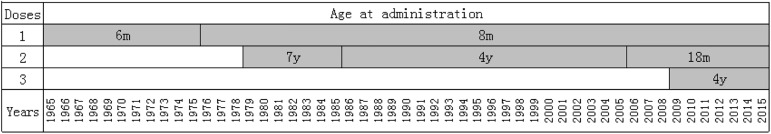
Measles vaccination strategy in Shanghai, 1965–2015.

Measles is a highly contagious acute viral illness that most countries worldwide have strived to eradicate [1–3]. Shanghai is located in eastern China, and has a highly developed economy. Measles vaccination began in Shanghai in 1965, making it one of the first cites to use the measles vaccine. The measles vaccination schedule has been adjusted several times over the past 50 years (Fig 1). Initially, the measles vaccine was administered as a single dose to children aged between 6 months and 6 years. The routine immunization of children against measles has been strengthened. At present, the vaccination schedule has been adjusted to 3 doses (at 8 months, 18 months, and 4 years of age), and periodic supplemental immunization was carried out annually for children less than 14 years of age.

Ongoing measles vaccination over the past 50 years has greatly reduced the incidence of measles in Shanghai. However, in recent years, a number of challenges have been encountered in the process of eliminating measles. First, the migrant population, mainly young and middle-aged adults, increased from 3.05 million in 2000 to 8.97 million in 2010. Further, the proportion of migrants increased from 18.2% in 2000 to 38.7% in 2010 [4]. The rapid increase in the migrant population changed the demographic structure in Shanghai. Second, the epidemiology of measles was changed; it is no longer dominated by pediatric cases. These problems are seen in other cities and regions as well [5,6]. Thus, we aim to describe the measles epidemic trend over the past 30 years in a central district in Shanghai, using historical surveillance data, which can help develop strategies of eliminating measles in developed cities.

## Methods

The study was conducted in the Jing’an District, which is located in the center of Shanghai. In 2015, the total geographic area of the Jing'an District was 7.6 km^2^ and the total population was 350 thousand. Measles were notifiable through the measles surveillance system. The historical surveillance data of measles was collected from 1984 to 2015. Measles case was diagnosed based on the standard case definitions in the Chinese Measles Surveillance Program. A suspected measles case was clinically diagnosed by physicians as one with all of the following clinical presentations: 1) fever; 2) maculopapular rash; and 3) cough, coryza or conjuctivities. A laboratory-confirmed case is a suspected case with positive measles-specific IgM antibodies (from 2001 to 2015) or hemaogglutination inhibition (HI) antibodies (from 1984 to 2000). A clinically diagnosed case is a suspected case without serological testing, but which was epidemiologically linked to a laboratory confirmed case. In this study, we used the data for all laboratory confirmed or clinically diagnosed measles cases. The collected information of measles cases included age, date of onset, and residence (local and migrant). Since the local residents and migrants were covered by different measles vaccination strategies and coverage in different areas in China, we calculated the incidence of measles for the local residents and migrants separately.

We classified all measles cases among local residents from 1984 to 2015 into 8 age groups: <1 year, 1–4 years, 5–9 years, 10–14 years, 15–19 years, 20–29 years, 30–39 years and 40–49 years. We also classified all measles cases among local residents into 5 birth cohorts according to the implementation of changes to the measles vaccination schedule in Shanghai. Group A: people born during 1959–1971; the first group of people who were vaccinated against measles with a single dose of the measles vaccine. Group B: people born during 1972–1981; this group was vaccinated against measles in two doses at 8 months and 7 years of age. Group C: people born during 1982–2014; this group was vaccinated against measles in at least two doses of the measles vaccine by the age of 4 years. Because of the large time span included in Group C, we further divided this group into 3 subgroups for analysis, each of which has a time span of approximately 10 years. Group C1: people born during 1982–1991; Group C2: people born during 1992–2001; Group C3: people born during 2002–2014.

Considering that the total number of measles cases was small, we divided the years 1984–2015 into 3 time periods: 1984–1994, 1995–2005, and 2006–2015. We then calculated the average annual incidence of measles in local residents in these 3 time periods by different age groups and birth cohorts (the total number of measles cases in local residents divided by the total number of local residents by age groups and birth cohorts in a given period) to understand the measles epidemic trend over the past 30 years in the Jing'an District. Since we lacked detailed information about migrants in terms of age groups or birth cohorts, we calculated only the overall average annual incidence of measles for migrants (the total number of measles cases in migrants devided by the total number of migrants in a given period).

We described the measles epidemic trend by calculating the incidence stratified by years, then we compared the age distribution of local residents and the migrants by Wilcoxon W rank sum test.

### Ethical consideration

This study was part of surveillance data analysis and was determined to be exempt from institutional review board review by the Jing’an District Center for Disease Control and Prevention. All data were kept confidential without patient identifiers. Cases were anonymised and identified with a unique study code instead of patient identifiers.

## Results

### 1. The distribution (time, age) of measles cases over the past 30 years

**Fig 2:**
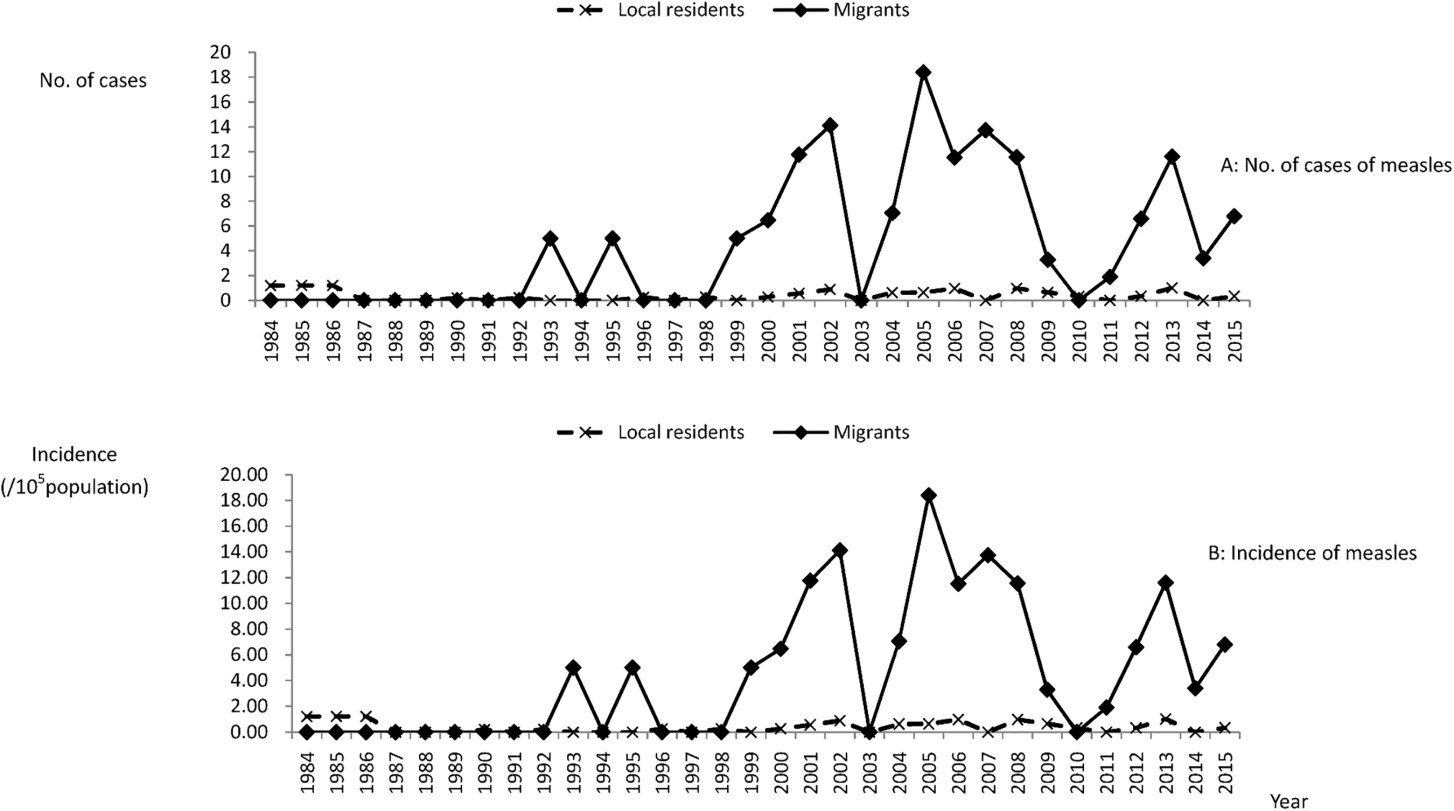
The number of cases and incidence of measles during 1984–2015.

A total of 103 measles cases were reported from the Jing’an District, Shanghai, from 1984 to 2015. Of them, 46 cases were of local residents and the other 57 cases were of migrants. Based on the epidemic curves and incidence by year (Fig 2), we determined that the number of measles cases per year decreased from 1987 to 1999, but rebounded after 2000. The majority of measles cases reported after 2000 were among migrants, which was different compared to before 2000. The incidence of measles among local residents was maintained at a relatively low level. From 1984 to 1986, the incidence of measles was 1.19 to 1.21 per 100,000 population, and then decreased during 1987–1999 to less than 0.3 per 100,000 population. The incidence increased slightly after 2000, reaching as high as 1.00 per 100,000 population in 2013. In the meantime, the incidence of measles among migrants significantly increased after 2000. From 2000 to 2015, measles cases were reported from migrants every year, except 2003 and 2011, with the incidence reaching as high as 18.40 per 100,000 population in 2005. From the epidemic curve of measles incidence, we found that the measles incidence of local residents always increased after that of the migrants (S1 and S2 Tables).

We divided the years 1984–2015 into 3 time periods. During 1984–1994, there was a single measles case reported among migrants in a 20-year-old patient, and 20 measles cases among local residents aged 6 months to 20 years, with a median age of 2.5 years. During 1995–2005, there were 25 cases among migrants aged 2 months to 30 years, with a median age of 4 years, and 12 cases among local residents aged 7 months to 44 years, with a median age of 28.5 years. The age distribution of patients with measles was statistically different between the local residents and migrants (Wilcoxon W rank sum test Z = 2.534, P = 0.011). During 2006–2015, there were 31 cases of measles among migrants aged 5 months to 44 years, with a median age of 21 years, and 14 cases among local residents aged 7 months to 46 years, with a median age of 39.5 years. The age distribution was not statistically different between the local residents and migrants for the 2006–2015 time period (Wilcoxon W rank sum test Z = 1.829, P = 0.067).

### 2. Incidence rate of measles in local residents by age group

**Fig 3:**
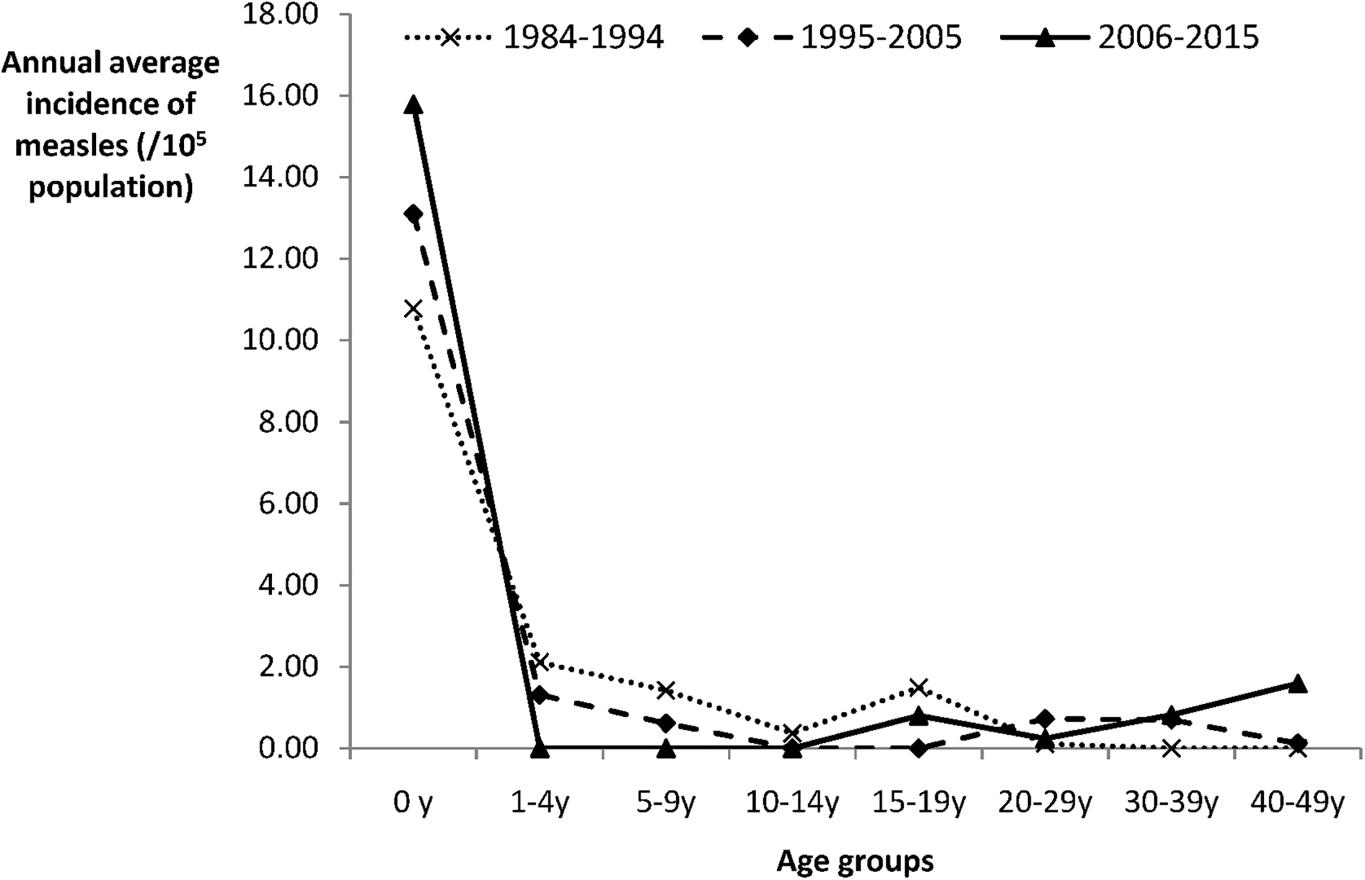
The incidence of measles in local residents by age group in 3 time periods.

The incidence of measles in local residents by age group in the 3 time periods is shown in Fig 3. We found that the trend of the measles incidence was different among the age groups. The infants less than 1 year of age still had the highest incidence of measles, and the incidence rose over the past 30 years. For children 1–14 years of age, the incidence continued to decline and became close to 0 in recent years. For the adults over 30 years of age, the incidence continuously rose from 0 over the past 30 years, but for adults 15–29 years of age, the incidence fluctuated (S1 and S2 Tables).

### 3. Incidence of measles in local residents by birth cohort

The incidence of measles in local residents by birth cohort is listed in Table 1. The trend of the measles incidence was different among the birth cohorts. For people born between 1959 and 1971, who were the first to be vaccinated against measles and received a single dose, the incidence of measles continuously rose over the past 30 years. For people born between 1972 and 1981 and those born between 1982 and 1991, who were vaccinated with at least 2 doses of the measles vaccine, the measles incidence rate initially declined but then rebounded. For people born between 1992 and 2014, we found a decline with no rebound in the measles incidence, likely because of the shorter observation period (S1 and S2 Tables).

**Table 1:** The incidence of measles in local residents by birth cohort in the Jing'an District during 1984–2015.

Birth cohorts*	Annual average incidence of measles (/100,000 population)		
	1984–1994	1995–2005	2006–2015
Group A	0.36	0.79	1.06
Group B	0.88	0.47	0.81
Group C1	2.60	0.00	0.49
Group C2	-	1.24	0.00
Group C3	-	7.67	1.62

*Group A: people born during 1959–1971, the first group of people who were vaccinated against measles with a single dose of the measles vaccine

Group B: people born during 1972–1981, who were vaccinated against measles in two doses at 8 months and 7 years of age

Group C: people born during 1982–2014, who received at least two doses of measles vaccine by the age of 4 years

Group C1: people born during 1982–1991

Group C2: people born during 1992–2001

Group C3: people born during 2002–2014

## Discussion

As one of the most developed cities in China, Shanghai has an advanced community health service system. Since the measles vaccine was first used in 1965, the vaccine coverage has remained at a relatively high level. Especially in recent years, the measles vaccine coverage has remained above 95% [7]. And according to the historical data, the reported measles vaccination coverage was always above 98% during 1984–2015 in Jing’an District. In the 1990s, the incidence of measles decreased to less than 1 per 100,000 population, close to complete eradication of measles [8]. At that time, there were only rare measles cases reported in the Jing’an District. However, after 2000, the incidence of measles rebounded in both the Jing’an District and Shanghai [9]. Many researchers have tried to find the cause of the rebound in measles in order to guide adjustment of the vaccination strategies. In our study, we analyzed the trend of the measles incidence in different groups based on original measles surveillance data.

Similar to the results of many other studies, the significant increase of the incidence of measles was mainly due to the high incidence of measles among migrants with low vaccine coverage [10–13]. The incidence of measles in migrants was several times higher than that of local residents in recent years in the Jing’an District. At the same time, the incidence of measles among the local population also rebounded, despite the fact that the measles vaccine coverage in this population was very high.

Since 2009, the measles vaccination schedule for children in Shanghai was adjusted to 8 months, 18 months and 4 years. Supplemental immunization was carried out every year for children less than 14 years of age in order to ensure the high vaccine coverage. Continuous strengthening of immunization strategies for children resulted in the decline in the incidence of measles in children 1–14 years of age among local residents over the past 30 years. In contrast, the incidence of measles among both infants below the age of 1 year and adults over the age of 30 years continued to increase over the past 30 years. This demonstrates that even if the measles vaccine immunization strategy for children is perfect, it is not sufficient to eliminate measles in the entire population.

A number of studies have reported changes in the epidemiological characteristics of measles after use of the measles vaccine, particularly the increasing proportion of adult and infants cases [5, 14–16]. In our study, based on relatively complete surveillance data over past 30 years in the Jing'an District, we calculated the change in the incidence of different groups within the population. From the age group analysis, children under the age of 1 year and adults over the age of 30 years had increasing incidence of measles. The maximum age of measles among adults has a tendency to increase, and measles cases among the elderly were reported several years earlier in local residents than in migrants. From the birth cohort analysis, the group of people who were vaccinated against measles first had a continuously increasing incidence of measles in recent years. In the other birth cohorts, the incidence of measles after vaccination declined initially, followed by a rebound. Together, these phenomena suggest that the population immunity against measles after vaccination gradually reduces with time. The younger the age when measles vaccination was initiated, the wider the age range affected by the immunity decline. It is believed that the increased incidence of measles in infants less than 1 year of age may be associated with the reduced immunity against measles among young adults of childbearing age [17–19].

Continuous strengthening of immunization strategies for children alone cannot achieve a sustained decline in the measles incidence of the whole population. The problem faced by Shanghai is faced by a number of developed cities. Even in the countries and regions where measles has been eliminated, similar morbidity characteristics of measles would be observed when migrants, who are not immunized to measles, transmit measles to the local population [20–23]. To address this problem, we call for an adult immunization program for the measles vaccine, especially among migrants. If adult immunization cannot be fully carried out, we can predict that the average and maximum age of people with measles will continue to increase along with the decline in the population immunity. This will result in cases of measles among the elderly, which could cause new challenges including severe cases of measles and death due to measles. At present, supplemental measles vaccination for adult migrants is partially implemented; however, the incidence of measles has not declined as expected. This suggests that measles vaccination rate for adult migrants still needs to improve.

The limitation of our study lies in the fact that the area of the Jing'an District is small and the population base is small, which affects the representativeness of the results. However, the epidemiological characteristics of measles in the Jing'an District have been consistent with most developed cities in recent years, and the complete historical surveillance data was available for the past 30 years, which allowed this study to be carried out.

In conclusion, with the widespread implementation of the measles vaccines, the incidence of measles in children has decreased significantly. However, the incidence of measles in elderly adults and infants increased in some developed regions, which slowed the progress of measles elimination. We recommend supplemental immunization against measles in adults to reduce immunity decline, especially among migrants.

## Supporting information

S1 Table Case list. Information of measles cases during 1984–2015 in Jing’an District, Shanghai.
(XLSX)

S2 Table Population number. The population number of local residents and migrants during 1984–2015 in Jing’an District, Shanghai.
(XLSX)
